# Effect of Obesity among Hospitalized Cancer Patients with or without COVID-19 on a National Level

**DOI:** 10.3390/cancers14225660

**Published:** 2022-11-17

**Authors:** Jonathan Cottenet, Solène Tapia, Patrick Arveux, Alain Bernard, Tienhan Sandrine Dabakuyo-Yonli, Catherine Quantin

**Affiliations:** 1 Biostatistics and Bioinformatics (DIM), University Hospital, BP 77908, 21079 Dijon, France; 2 University of Bourgogne Franche-Comté, 21000 Dijon, France; 3 Center for Primary Care and Public Health, Unisanté, University of Lausanne, 1015 Lausanne, Switzerland; 4 Department of Thoracic and Cardiovascular Surgery, Dijon-Bourgogne University Hospital, 14 Rue Gaffarel, BP 77908, 21079 Dijon, France; 5 Breast and Gynaecologic Cancer Registry of Côte d’Or/Epidemiology and Quality of Life Research Unit, Georges François Leclerc Centre-UNICANCER, 1 Rue Professeur Marion, 21000 Dijon, France; 6 Lipids, Nutrition, Cancer Research Center, INSERM U1231, 21000 Dijon, France; 7 Inserm, CIC 1432, 21000 Dijon, France; 8 Clinical Epidemiology/Clinical Trials Unit, Dijon-Bourgogne University Hospital, Clinical Investigation Center, 21000 Dijon, France; 9 Inserm, High-Dimensional Biostatistics for Drug Safety and Genomics, Le Centre de Recherche en Epidémiologie et Santé des Populations (CESP), Université Paris-Saclay (UVSQ), 94800 Villejuif, France

**Keywords:** obesity, COVID-19, cancer, tumor subtype, mortality, intensive care unit, medico-administrative data, SARS-CoV-2, France

## Abstract

**Simple Summary:**

Few papers have looked for an association between obesity and mortality risk in cancer patients with COVID-19 but, to our knowledge, none have studied this association in relation to the severity of obesity. We performed a study using data from the French national hospital database to study the effect of obesity (and its severity) on the risk of intensive care unit (ICU) admission, severe complications, and in-hospital mortality in cancer patients hospitalized for COVID-19 or not. The risk of ICU admission or severe complications was higher in cancer patients with obesity compared to cancer patients without obesity, regardless of cancer type and obesity severity. We did not find an excess obesity-related risk for in-hospital mortality, except for massive obesity in COVID-19 patients with hematological cancer and in non-COVID-19 patients with solid cancer. Further studies are warranted to better understand the relationship between obesity, and especially massive obesity, the prognosis of SARS-CoV-2 infection in cancer patients.

**Abstract:**

Cancer and obesity are well-known prognostic factors in COVID-19. Our objective was to study the effect of obesity (and its severity) on the risk of intensive care unit (ICU) admission, severe complications, and in-hospital mortality, in a population of cancer patients hospitalized with or without COVID-19. All patients hospitalized in France for cancer from 1 March 2020 to 28 February 2022 were included from the French national administrative database. The effect of obesity was estimated in COVID-19 and in non-COVID-19 cancer patients using logistic and survival regressions, taking into account age, sex, comorbidities, and different types of cancer. Among the 992,899 cancer patients, we identified 53,090 patients with COVID-19 (5.35%), of which 3260 were obese (6.1%). After adjustment, for patients with or without COVID-19, there is an increased risk of ICU admission or severe complications in obese patients, regardless of the type of obesity. Regarding in-hospital mortality, there is no excess risk associated with overall obesity. However, massive obesity appears to be associated with an increased risk of in-hospital mortality, with a significantly stronger effect in solid cancer patients without COVID-19 and a significantly stronger effect in hematological cancer patients with COVID-19. This study showed that in France, among hospitalized patients with cancer and with or without COVID-19, increased vigilance is needed for obese patients, both in epidemic and non-epidemic periods. This vigilance should be further strengthened in patients with massive obesity for whom the risk of in-hospital mortality is higher, particularly in epidemic periods for patients with hematological cancers.

## 1. Introduction

The World Health Organization declared a global pandemic in March 2020 [1] as a result of the severe acute respiratory syndrome coronavirus 2 (SARS-CoV2) outbreak. The 2019 coronavirus disease (COVID-19), caused by SARS-CoV-2, is still present and continues to cause significant mortality and morbidity worldwide. Indeed, COVID-19, as an extremely communicable virus, displays a range of conditions from no symptoms at all to severe illness and death [2,3,4,5], which has led to a shift in the focus of healthcare systems. As a result of health recommendations and changes in patient behavior, the pandemic also had indirect effects on morbidity and mortality

As of 28 January 2021, the COVID-19 epidemic was responsible for 99,727,853 confirmed cases worldwide, including 32,218,360 in Europe, and 2,137,670 deaths worldwide, 166,613 of which were in Europe [6]. In France, there were 3,053,617 confirmed cases and 73,049 deaths [7]. Based on expert opinion and data from the literature [8,9,10,11,12], the Haut Conseil de la Santé Publique (HCSP) considers that several factors can lead to severe SARS-Cov-2 infection. These include cardiovascular disease, diabetes (including unbalanced insulin-dependent diabetes or diabetes with secondary complications), chronic respiratory disease, and patients with congenital or acquired immunosuppression. In particular, patients treated for cancer (e.g., cancer chemotherapy, immunosuppressive therapy, biotherapy) are at increased risk of developing a severe form. People with morbid obesity are also cited as being at higher risk [13,14,15,16]. 

Cancer patients, due to the disease and specific treatments such as chemotherapy or surgery, are considered immunocompromised and therefore have a higher risk of infection [17,18,19,20]. Several publications have shown that cancer patients are three times more likely to develop serious complications from COVID-19 [9,10,11,15,21]. More specifically, in a study focused on lung cancer [22], we showed that, in this population, SARS-CoV-2 was associated with a 7-fold increased risk of in-hospital mortality and an almost 5-fold increased risk of serious complications. 

Obesity is also a well-known prognostic factor in COVID-19. In numerous studies, obesity is associated with increased morbidity and mortality in patients with COVID-19 [23,24,25,26,27]. However, in some populations, there seems to be an obesity paradox, especially in severely ill populations such as respiratory failure or cancer patients [23,28,29,30,31,32,33]. Indeed, it appears that in these studies, obesity was not significantly associated with increased morbidity and mortality in cancer patients with COVID-19. Some studies even found that the risk of mortality was lower in cancer patients with metabolic comorbidities. However, the controversy persists as most of these studies were small and may have lacked power. Moreover, they did not take into account the different waves of the epidemic and more specifically 2021 and/or 2022. 

There are very few data describing the natural history of cancer patients with COVID-19. Some papers have looked for an association between obesity and mortality risk but, to our knowledge, none have studied this association in relation to the severity of obesity. We hypothesized that there may be an intercorrelated relationship between obesity and cancer and that their mutual pathogenetic attributes may predispose individuals to a different prognosis when they develop a SARS-CoV-2 infection. 

Using data from the French national hospital database, which includes more than 1,000,000 patients hospitalized for cancer between March 2020 and March 2022, our aim was, therefore, to investigate the effect of obesity (and its severity) on the risk of intensive care unit (ICU) admission, severe complications and in-hospital mortality in cancer patients hospitalized with or without COVID-19.

## 2. Materials and Methods

### 2.1. Database

We conducted a retrospective cohort study using the national hospital Programme de Médicalisation des Systèmes d’Information (PMSI) database, which is designed to include discharge abstracts for all inpatient admissions to public and private hospitals in France. Inspired by the American DRG (diagnosis-related groups) model, the information in these abstracts is anonymous and covers both medical and administrative data. Diagnoses identified during the hospital stay are coded according to the 10th edition of the International Classification of Diseases (ICD-10), and procedures performed during hospitalization are coded according to the French Common Classification of Medical Procedures. The fact that these national data are used for the allocation of hospital budgets encourages improvement in data quality in terms of coherence, accuracy, and exhaustiveness. Therefore, these hospital data have been used in medical research for many years [34,35,36,37,38,39,40,41,42,43,44], and their quality has been confirmed in recent studies on COVID-19 [5,45,46,47,48,49,50].

### 2.2. Population

We included all patients hospitalized for or with cancer from 1 March 2020 to 28 February 2022. Hospital stays of more than one day (i.e., excluding a day of hospitalization, such as a chemotherapy session) for cancer were identified using the International Classification of Diseases, 10th Revision (ICD-10) codes, via principal diagnoses (PD), related diagnoses (RD) or associated diagnoses (AD). It should be noted that only the 1st stay identified during the inclusion period has been retained so that a patient is counted only once. We considered all cancers corresponding to malignant tumors (all those beginning with ‘C’, Table S1), with and without metastases, and separated them into different tumor subtypes (solid cancers with metastases, solid cancers with localized tumor, and hematological cancers). 

Among these patients, we separated a group of patients with mention of COVID-19 at the time of oncology stay, and another group of patients without mention of COVID-19 either at the time of inclusion or during the whole study period. COVID-19 patients were identified using ICD-10 codes (U0710, U0711, U0712, U0714 or U0715). We also identified obese patients at the time of oncology stay using ICD-10 codes E66 (except overweight) in PD, RD, or AD. The diagnosis of obesity was based on a body mass index (BMI) of ≥30 kg/m^2^. Obesity was also classified according to its severity: massive obesity (BMI ≥ 50 kg/m^2^), morbid obesity (BMI 40 to 49 kg/m^2^), and standard obesity (BMI 30 to 39 kg/m^2^). The classification of the ICD-10 codes used is given in Table S2.

### 2.3. Outcomes 

Our primary outcome was transferring to the intensive care unit (ICU), which was determined by the presence of an ICU stay indicator in the filed claims during the cancer stay. Our second outcome was severe complications (including acute respiratory and kidney diseases, stroke, myocardial infarction, atrial fibrillation, and venous thrombosis including pulmonary embolism) during the cancer stay and 90 days after discharge. Finally, our third outcome was in-hospital mortality, defined as any patient who died in hospital during the cancer stay and within 90 days after discharge.

### 2.4. Variables 

Patient characteristics such as age (seven age classes were defined: ≤40, 41–50, 51–60, 61–70, 71–80, 81–90, and >90) and gender were also retrieved. We also extracted all diagnoses from the discharge abstracts to retrieve patients’ comorbidities: hypertension, diabetes, dementia, HIV, heart failure, chronic respiratory and renal diseases, cirrhosis, peripheral vascular disease, dyslipidemia, deficiency anemia, and pulmonary bacterial infection. Finally, we identified chemotherapy in the previous 2 years and in the month following the inclusion stay, via ICD-10 code Z511. 

### 2.5. Statistical Analysis

Qualitative variables are provided as frequencies (percentages) and were compared using the Chi-2 test or Fisher’s exact test. Quantitative variables are provided as means ± standard deviation (SD) and medians [interquartile range (Q1–Q3)], and were compared using the Student’s *t* test or Mann-Whitney test. 

Our outcomes were compared for all patients and then according to the type of tumor (solid cancers with metastasis, with localized tumors, hematological cancers) and the type of obesity (massive, morbid, standard). 

To estimate the effect of obesity in cancer patients hospitalized depending on the COVID-19 infection, all the following models were performed in both COVID-19 and non-COVID-19 patients, after adjustment on age, sex, chemotherapy, and other comorbidities:

- Logistic regression models to estimate the effect of obesity on the risk of transfer to ICU, severe complications, and in-hospital mortality at inclusion.
- Fine and Gray models to estimate the effect of obesity on the risk of severe complications within 90 days after discharge. This model takes into account the competing risk between severe complications and in-hospital mortality, as, death may prevent the observation of severe complications.
- Cox models to estimate the effect of obesity on the risk of in-hospital mortality within 90 days after discharge.

We also performed sensitivity analyses by separating the analyses into two different periods of the epidemic corresponding to two different viral periods: March–December 2020 (the original virus) and January 2021–February 2022 (the emergence of the alpha variant in France in late 2020).

The statistical significance threshold was set at < 0.05. All analyses were performed using SAS (SAS Institute Inc, Version 9.4, Cary, NC, USA).

## 3. Results

### 3.1. Patient Characteristics

We included 922,899 patients diagnosed with cancer, among whom we identified 53,090 patients with COVID-19 (5.35%). Among these patients, 3260 were classified as obese (6.14%) (Table 1). 

In cancer patients with COVID-19 (Table 1), the rate of men was lower in obese than in non-obese patients (51.99% vs. 60.08%, *p* < 0.01), and obese patients were younger than non-obese patients.

In addition, the rates of the majority of comorbidities studied were higher in obese patients than in non-obese patients, except for dementia and HIV (Table 1). Finally, regarding outcomes (Table 1), the rate of ICU admission was two times higher in obese than in non-obese patients (30.4% vs. 13.6%, *p* < 0.01). The rate of severe complications was also higher whether at inclusion (79.4% vs. 67.4, *p* < 0.01) or within 90 days (83.6% vs. 73.4%, *p* < 0.01). In contrast, the in-hospital mortality rate was lower in obese patients than in non-obese patients whether at inclusion (24.7% vs. 30.7%, *p* < 0.01) or within 90 days (30.1% vs. 38.9%, *p* < 0.01).

Among obese patients (N = 3260), 83% were classified as standard obesity (N = 2704), 15% as morbid obesity (N = 497), and 2% as massive obesity (N = 59). Regardless of the type of obesity, patients were younger than non-obese patients and less often male (Table 1). It should be noted that men represent only 30% of the patients with morbid or massive obesity. Concerning comorbidities, the majority of comorbidities studied were significantly higher in standard obese patients than in non-obese patients, except for dementia and HIV (Table 1). We found the same results for morbidly obese patients even if the discrepancy was not significant for cirrhosis, peripheral vascular disease, deficiency anemia, and pulmonary bacterial infection. For the comparison between non-obese patients and those with massive obesity, we found that the rates of hypertension, chronic respiratory and kidney disease, cirrhosis, and diabetes were significantly higher in massively obese patients.

Whatever the type of obesity, the rate of ICU admission or severe complications was higher in obese patients than in non-obese patients (Table 1). Regarding in-hospital mortality, rates were significantly lower in patients with standard or morbid obesity than in non-obese patients, either at inclusion (respectively 24.2%, 26.6%, and 30.7%) or at 90 days (29.6%, 31.4%, and 38.9%). In contrast, the rates were higher in massively obese patients, although this difference was not significant, either at inclusion (33.9% vs. 30.7%, *p* = 0.60) or at 90 days (40.7% vs. 38.9%, *p* = 0.78).

For non-COVID-19 patients, we found similar results regarding patient characteristics to those for COVID-19 patients (Table S1).

### 3.2. Outcomes Depending on the Type of Tumor

When we look at the type of tumor (Table 2), the rate of hematological cancer was similar between obese and non-obese patients (26.29% vs. 25.45%, *p* = 0.29). However, the rate of solid cancer with metastasis was higher in non-obese patients (39.67% vs. 31.07%, *p* < 0.01), while the rate of solid cancer with localized tumor was higher in obese patients (42.64% vs. 34.88%, *p* < 0.01). The same results were found whatever the type of obesity, although the differences were not significant for patients with massive obesity (Table 2). 

Regarding the description of the type of cancer, among non-COVID-19 patients (Table S2), the rate of patients with hematological cancer or solid cancer with metastasis was lower in obese patients than in non-obese patients (8.49% vs. 10.45% for hematological cancer and 24.62% vs. 29.69% for solid cancer with metastasis). Furthermore, it should be noted that among non-COVID-19 patients with massive obesity, the rate of patients with hematological cancer was similar to that in patients without obesity (11% vs. 10.45 %, *p* = 0.58). Conversely, and as observed in the population of patients with cancer and COVID-19, the rate of solid cancer with localized tumors was higher in obese patients (whatever the type of obesity).

Concerning our outcomes, for COVID-19 patients, whatever the type of tumor and the type of obesity, the rate of ICU admission or severe complications was higher in obese patients than in non-obese patients (Table 3). 

However, the in-hospital mortality rate appears to differ according to the type of tumor and the type of obesity (Table 3). In hematological cancer, the more severe the obesity, the higher the mortality rate. Compared to non-obese patients, the mortality rate is lower for standard obese patients, similar for morbidly obese patients, and higher for massive obese patients. In solid cancer with metastasis, compared to non-obese patients, the mortality rate is lower for standard obese patients and massively obese patients, while it is equivalent for morbidly obese patients. In solid cancer with localized tumors, compared to non-obese patients, the mortality rate is lower for standard obese patients and morbidly obese patients, while it is equivalent massively obese patients.

Compared to the COVID-19 population, the results are similar for ICU admissions or complications in the non-COVID-19 population but are not the same for in-hospital mortality, especially in the population with massive obesity (Table S3). Thus, for patients with hematological cancer, the mortality rate for massive obesity is similar to that of patients without obesity. For patients with solid cancers (with metastases or with localized tumors), the in-hospital mortality rate is higher in patients with massive obesity than in patients without obesity.

### 3.3. Multivariate Analyses

Regarding the effect of obesity on COVID-19 cancer patients, after adjusting for age, sex, chemotherapy, and comorbidities, we found that those with obesity have twice the risk of admission to ICU compared to those without obesity, regardless of cancer type (Table 4). We found the same results concerning the risk of severe complications, especially for severe complications during the admission stay (Table 4). With regard to hospital mortality, there was no excess risk linked to obesity, in cancer patients with COVID-19 (Table 4), regardless of cancer type.

In the multivariate analyses for the non-COVID-19 patients (Table S4), we found the same results by type of cancer and in COVID-19 patients, i.e., obesity was associated with an increased risk of ICU admission and severe complications, but not with an increased risk of hospital mortality.

For COVID-19 patients, if we focus on the effect according to the severity of the obesity, we found that the risk of admission to ICU was two times higher whatever the severity and whatever the type of cancer (Table 5), even if this risk was not significant for massive obese patients in hematological cancer and solid cancer with metastasis. The risk of severe complications within 90 days was also higher, although not significant, for patients with solid cancer with metastasis and morbid or massive obesity (Table 5).

Finally, concerning in-hospital mortality (Table 5), massive obesity seems to be a risk factor, particularly in patients with hematological cancer, where this risk is significant (OR = 3.09 [1.12–8.57] during the inclusion stay and HR = 2.20 [1.18–4.10] within 90 days). For the other types of obesity (standard or morbid), there was no excess risk due to obesity in patients with solid cancer (with metastasis or with localized tumor).

In the non-COVID-19 population, after looking at the type of obesity, we still found that the risk of admission to intensive care was higher for all types of obesity and all types of cancer (Table S5). We found the same results for the excess risk of severe complications. Regarding in-hospital mortality, massive obesity again seems to be a risk factor, and even more so in patients with solid cancer (with metastasis or with localized tumor).

In the sensitivity analyses, separating the analyses according to the health conditions of the pandemic in France, we found the same trends as in the results above, whatever the period considered (2020 or 2021–2022).

## 4. Discussion

Our results showed that in France, for patients with or without COVID-19, there is an increased risk of ICU admission or severe complications in obese patients, regardless of the type of obesity. Regarding in-hospital mortality, there is no excess risk associated with overall obesity. However, massive obesity appears to be associated with an increased risk of in-hospital mortality, with a significantly stronger effect in solid cancer patients without COVID-19 and a significantly stronger effect in hematological cancer patients with COVID-19. We also found an increased risk of in-hospital mortality in solid cancer patients with localized tumors among COVID-19 patients, but this result was not significant. However, we can hypothesize a lack of power in this subgroup with few patients with massive obesity. Focusing on COVID-19, cancer patients with obesity are twice as likely to be admitted to ICU compared to cancer patients without obesity, regardless of cancer type and obesity severity. We found the same results for the risk of severe complications. However, surprisingly, we did not find a significant excess obesity-related risk for in-hospital mortality among cancer patients hospitalized for COVID-19, except for massive obesity in patients with hematological cancer. 

The fact that an increased risk of complications and ICU admissions were shown in obese cancer patients was to be expected. Indeed, many studies have shown similar results in the general population and cancer patients.

However, we did not find that obesity had a negative effect on in-hospital mortality in cancer patients hospitalized for COVID-19. While this result may seem surprising since obesity is classically considered as contributing to a worse prognosis in the COVID-19 population [23,24,25,26,27], it is consistent with other studies [23,28,29,30,31,32,33,51] in cancer patients which did not find that mortality was increased (most ORs were close to 1 even after adjustment). This is the case, for example, for the cancer consortium cohort study, which found that obesity was not significantly associated with the risk of 30-day mortality, either in univariate analysis or after adjustment for age, sex, and smoking (OR = 0.99 [0.58–1.71]). This result is also consistent with a meta-analysis including 2117 mixed ambulatory and hospitalized cancer patients (OR = 0.92 [0.66–1.28]. Secondly, it is well known that obese patients may paradoxically have lower mortality than non-obese patients [23]. 

This “obesity paradox” is observed in several epidemiological studies of chronic diseases, acute illnesses, and cancer, where mortality in non-obese patients is higher than in obese patients [52,53,54,55]. This phenomenon was also observed during the COVID-19 pandemic and has been supported in the US, using a mathematical model [56]. The lean mass found in obese people could be a possible explanation for the obesity paradox. Indeed, even if the increase in BMI is mainly due to fat mass, obese people have an increase in lean mass, including muscle mass, in particular cardiac muscle mass. The obesity paradox could therefore be linked to a protective effect of increased lean mass. [52]. According to Park et al., “the leading hypothesis for this phenomenon points to the greater metabolic reserve represented by the abundant adipose tissue, which enables the patient to withstand the course of severe acute or chronic illness”. 

Of course, the obesity paradox cannot be the only reason associated with these results. Indeed, in the most advanced stages, a characteristic feature of cancer is cachexia. Cancer cachexia is a multifactorial syndrome that leads to substantial weight loss, primarily due to skeletal muscle loss, and can have a substantially negative impact on the response to immune checkpoint inhibitors therapy, particularly inpatients with advanced non-small cell lung cancer [57]. Furthermore, cachexia is frequently obscured by obesity, leading to underdiagnosis and excess mortality [58]. In particular, Martin et al. showed that skeletal muscle depletion is a powerful prognostic factor of mortality, independent of body mass index [59]. However, we cannot reliably identify cachexia in our database. This is not necessarily surprising since cachexia is usually not recorded in hospital cohorts. Moreover, there still exists a gap in the clinical management of cachexia due to the complex nature of the condition, which may affect the ability to identify cachectic patients and appropriate treatment, for which there is no globally recognized ‘gold standard’ [60]. Therefore, cachexia remains a largely underestimated condition.

Considering the severity of obesity, compared to patients without obesity, we did not find an excess mortality risk for patients with standard or with morbid obesity. This result is consistent with other studies looking at this impact on COVID-19 patients and chronic illnesses [18,61,62]. Again, this may be related to the obesity paradox. Indeed, studies confirming the “obesity paradox” showed that patients with standard or morbid BMI had severe symptoms that led to admission to intensive care, but not to increased mortality. However, other papers have shown that the risk is increased for both the general and COVID-19 populations [63,64,65]. To our knowledge, this is the first study to show the effect of massive obesity in cancer patients with COVID-19, as we did not find any study that took into account a BMI of more than 50 kg/m2. We found an excess risk for all types of cancer, but this excess risk is in fact significant in patients with hematological cancer (this type of cancer is particularly associated with a severe form of COVID-19) [63,66,67], even if our number of patients is small. Further studies are thus needed to investigate the relationship between massive obesity and mortality in patients with cancer and COVID-19 infection. 

Not all cancer patients have the same risk of developing severe COVID-19. Indeed, the mechanisms underlying the progression of COVID-19 to a severe form include host factors such as age or cachexia, hypercoagulable states caused by cancer or drugs, and possible hyperexpression of entry factors such as angiotensin-2 converting enzyme or neurophilin-1 [68]. Other factors affecting viral immunity include myeloid cell dysfunction and cancer-related T-cell depletion [69]. The immune system is also involved in the virus-induced cytokine storm [70], which may be tempered or delayed by cancer- or drug-related immune depression [71]. For example, in a previous study [19], we found that among COVID cancer patients, those with hematological cancer had a slight risk of developing more complications during their stay than those with solid tumors. This could be explained by the treatment of these patients and the resulting immunosuppression during and after treatment, and probably throughout their lives. [68,69,70,71]. However, our data show that the effects of obesity on severe complications, admission to ICU, or mortality are nearly the same whatever the type of cancer and notably those with the worst prognosis such as hematological cancers and metastatic solid tumors. These results suggest that the influence of obesity on the prognosis of SARS-CoV-2 infection may be independent of the type of cancer, which may also seem surprising. Further studies are needed to further investigate the association between obesity and the prognosis of COVID-19 infection, regardless of cancer type. 

### 4.1. Strengths

In France, the national hospital database includes information from all French private and public hospitals, including data on COVID-19 patients and cancer patients. The fact that these national data are used for hospital budget allocation encourages high levels of data consistency, accuracy, and completeness. As a result, this study includes national data for more than 40,000 cancer patients and COVID-19, making it one of the largest studies in terms of the number of patients included and the completeness of the data. This study is also one of the few to provide data with full hospital follow-up, including all in-hospital mortality, regardless of the length of stay, and 90 days of follow-up. In addition, we were able to obtain information on the metastatic status of all solid tumors as well as on the patients’ comorbidities. We were also able to separate the different severities of obesity in our analyses by considering three categories (standard, morbid, and massive) and to our knowledge, none have studied this association in relation to the severity of obesity in cancer patients with COVID-19. We also considered two different periods of the epidemic corresponding to two different viral periods: the original virus and the emergence of the alpha variant in France in late 2020. 

### 4.2. Limitations

We recognize that there are several limitations. First, we included only hospitalized patients and we only measured hospital mortality, even if mortality in France mainly occurs in hospitals. Thus we cannot discount that a number of patients infected with COVID-19 were not hospitalized. Our study thus included only severe cases of COVID-19. Secondly, the data used to identify obesity are based on ICD-10 codes identified in the PMSI hospital database, and these data are collected for medico-economic purposes. However, we can assume that the completeness is satisfactory because hospitals have a strong financial incentive to collect these data. Indeed, the information is sometimes not filled in the discharge abstract when there is no impact on the patient’s care, which is often the case for ambulatory care, but this was not the case for the hospitalizations considered. We cannot ignore a misclassification between our different obesity groups. We may also have a lack of power in the massive obesity group, although we do have significant results for hematological cancers.

Given the reliance on ICD-10 codes for the selection of patients and the ascertainment of outcomes, there was a potential for misclassification-related or under-detection-related bias, especially for comorbidities [72,73]. Coding practices may vary among institutions as the people who perform the coding of diagnoses can be clinicians or information system technicians. Nevertheless, the quality of coding is checked in a standardized way by medical information professionals in each hospital in order to correct diagnoses (internal quality assessment), and the level of recording of co-morbidities has increased significantly in recent years, following its impact on the tariff of hospital stays. Because of the impact on hospital budget allocation, it has been shown that hospital claims data are becoming more accurate. In addition, a national external quality assessment program has been implemented to verify the quality of discharge abstracts in each hospital. Moreover, the use of hospital data to identify all hospitalized cancer cases may be questionable. It seems unlikely that cancer was not recorded in the PMSI data, as cancer is a serious condition that is difficult to ignore when summarizing a patient’s history as the coding of cancer has an impact on the hospital’s budget allocation.

In addition, we had no information on the stage of cancer or on the treatments, whether anti-cancer treatments (which would have allowed us to evaluate the potentially induced immuno-suppression) such as hormonal therapy, or other treatments used in other pathologies, which may influence (for good or bad) the severity of COVID-19 in hospitalized patients, such as different inhibitors (tyrosine kinase, IL6, ARA2). Finally, we do not have access to vaccination data, but cancer patients were a priority in the French regulations and the rate of completion of the initial vaccination schedule was over 90% for these patients in 2022.

## 5. Conclusions

This study showed that in France, among hospitalized patients with cancer and with or without COVID-19, increased vigilance is needed for obese patients, both in epidemic and non-epidemic periods. This vigilance should be further strengthened in patients with massive obesity for whom the risk of in-hospital mortality is higher, particularly in epidemic periods for patients with hematological cancers. 

Focusing on COVID-19, obese patients have an increased risk of admission to the ICU (a two-fold increase) and severe complications compared to non-obese patients. Surprisingly, we did not find any excess risk related to obesity regarding in-hospital mortality, suggesting that the paradigm of the “obesity paradox” may also apply to this population. Considering the severity of obesity, compared to patients without obesity, we did not find an excess mortality risk for patients with standard or morbid obesity. However, to our knowledge, this is the first study to show an effect the massive obesity, especially in hematological cancer patients.

This study also provides information about the role of obesity according to the different types of cancer for which the prognosis is worse such as hematological cancers and all metastatic cancers. We found nearly the same results as described above, whatever the type of cancer. These results suggest that the influence of obesity on the prognosis of SARS-CoV-2 infection may be independent of the type of cancer. Further studies are warranted to better understand the relationship between obesity, especially massive obesity, and the prognosis of SARS-CoV-2 infection in cancer patients.

## Figures and Tables

**Table 1:** Characteristics of COVID-19 cancer patients by obesity.

	No Obesity (1)	Obesity (2)	*p*-Value (1 vs. 2)	Standard Obesity (3)	Morbid Obesity (4)	Massive Obesity (5)	*p*-Value (1 vs. 3)	*p*-Value (1 vs. 4)	*p*-Value (1 vs. 5)
N	49,830	3260		2704	497	59			
Men, *n*(%)	29,939 (60.08)	1695 (51.99)	<0.01	1494 (55.25)	182 (36.62)	19 (32.20)	<0.01	<0.01	<0.01
Age, mean (std)	72.58 (14.32)	68.99 (11.61)	<0.01	69.43 (11.55)	67.32 (11.64)	62.88 (11.13)	<0.01	<0.01	<0.01
Age group (years)			<0.01				<0.01	<0.01	<0.01
≤40	1379 (2.77)	55 (1.69)		40 (1.48)	12 (2.41)	3 (5.08)			
41–50	1817 (3.65)	158 (4.85)		124 (4.59)	28 (5.63)	6 (10.17)			
51–80	30,644 (61.50)	2550 (78.22)		2105 (77.85)	396 (79.68)	49 (83.05)			
81–90	12,846 (25.78)	443 (13.59)		386 (14.28)	56 (11.27)	1 (1.69)			
>90	3144 (6.31)	54 (1.66)		49 (1.81)	5 (1.01)	0			
Chemotherapy, n(%)	22,967 (46.09)	1207 (37.02)	<0.01	1009 (37.32)	174 (35.01)	24 (40.68)	<0.01	<0.01	0.40
Comorbidities, n(%)									
Hypertension	16,747 (33.61)	1869 (57.33)	<0.01	1536 (56.80)	296 (59.56)	37 (62.71)	<0.01	<0.01	<0.01
Dementia	2612 (5.24)	81 (2.48)	<0.01	68 (2.51)	13 (2.62)	0	<0.01	<0.01	0.08
HIV	191 (0.38)	7 (0.21)	0.13	6 (0.22)	1 (0.20)	0	0.18	1	1
Heart failure	4467 (8.96)	416 (12.76)	<0.01	316 (11.69)	91 (18.31)	9 (15.25)	<0.01	<0.01	0.09
Chronic respiratory disease	924 (1.85)	154 (4.72)	<0.01	112 (4.14)	38 (7.65)	4 (6.78)	<0.01	<0.01	0.02
Chronic kidney disease	4767 (9.57)	438 (13.44)	<0.01	363 (13.42)	74 (14.89)	1 (1.69)	<0.01	<0.01	0.04
Cirrhosis	1114 (2.24)	133 (4.08)	<0.01	112 (4.14)	17 (3.42)	4 (6.78)	<0.01	0.08	0.04
Diabetes	9275 (18.61)	1320 (40.49)	<0.01	1064 (39.35)	234 (47.08)	22 (37.29)	<0.01	<0.01	<0.01
Peripheral vascular disease	2315 (4.65)	196 (6.01)	<0.01	166 (6.14)	29 (5.84)	1 (1.69)	<0.01	0.21	0.53
Dyslipidemia	2533 (5.08)	379 (11.63)	<0.01	315 (11.65)	59 (11.87)	5 (8.47)	<0.01	<0.01	0.23
Deficiency Anemia	2881 (5.78)	223 (6.84)	0.01	186 (6.88)	33 (6.64)	4 (6.78)	0.02	0.42	0.78
COPD	3674 (7.37)	362 (11.10)	<0.01	300 (11.09)	56 (11.27)	6 (10.17)	<0.01	0.001	0.45
Pulmonary bacterial infection	3149 (6.32)	379 (11.63)	<0.01	331 (12.24)	41 (8.25)	7 (11.86)	<0.01	0.08	0.10
Outcomes, n(%)									
Admission to ICU	6753 (13.55)	992 (30.43)	<0.01	825 (30.51)	146 (29.38)	21 (35.59)	<0.01	<0.01	<0.01
Severe complication during the inclusion stay	33,599 (67.43)	2589 (79.42)	<0.01	2154 (79.66)	385 (77.46)	50 (84.75)	<0.01	<0.01	<0.01
In-hospital mortality during the inclusion stay	15,313 (30.73)	805 (24.69)	<0.01	653 (24.15)	132 (26.56)	20 (33.90)	<0.01	0.04	0.60
Severe complication within 90 days	36,583 (73.42)	2724 (83.56)	<0.01	2262 (83.65)	409 (82.29)	53 (89.83)	<0.01	<0.01	<0.01
In-hospital mortality within 90 days	19,377 (38.89)	981 (30.09)	<0.01	801 (29.62)	156 (31.39)	24 (40.68)	<0.01	<0.01	0.78

**Table 2:** Type of cancer by obesity among COVID-19 cancer patients.

	No Obesity (1)	Obesity (2)	*p*-Value (1 vs. 2)	Standard Obesity (3)	Morbid Obesity (4)	Massive Obesity (5)	*p*-Value (1 vs. 3)	*p*-Value (1 vs. 4)	*p*-Value (1 vs. 5)
N	49,830	3260		2704	497	59			
Hematological cancer, n(%)	12,682 (25.45)	857 (26.29)	0.29	731 (27.03)	110 (22.13)	16 (27.12)	0.07	0.09	0.77
Solid Cancer with metastasis, n(%)	19,767 (39.67)	1013 (31.07)	<0.01	840 (31.07)	156 (31.39)	17 (28.81)	<0.01	<0.01	0.09
Solid Cancer with localized tumor, n(%)	17,381 (34.88)	1390 (42.64)	<0.01	1133 (41.90)	231 (46.48)	26 (44.07)	<0.01	<0.01	0.14

**Table 3:** Admission to ICU, severe complication and in-hospital mortality of COVID-19 cancer patients by type of cancer and obesity.

**Hematological Cancer**	**No Obesity (1)**	**Obesity** **(2)**	***p*-Value** **(1 vs. 2)**	**Standard Obesity** **(3)**	**Morbid Obesity** **(4)**	**Massive Obesity (5)**	***p*-Value** **(1 vs. 3)**	***p*-Value** **(1 vs. 4)**	***p*-Value** **(1 vs. 5)**
	12,682	857		731	110	16			
Admission to ICU	2770 (21.84)	349 (40.72)	<0.01	295 (40.36)	48 (43.64)	6 (37.50)	<0.01	<0.01	0.14
Severe complication during the inclusion stay	8975 (70.77)	703 (82.03)	<0.01	593 (81.12)	94 (85.45)	16 (100)	<0.01	<0.01	0.01
In-hospital mortality during the inclusion stay	3798 (29.95)	238 (27.77)	0.18	196 (26.81)	34 (30.91)	8 (50)	0.07	0.83	0.10
Severe complication within 90 days	9735 (76.76)	730 (85.18)	<0.01	618 (84.54)	96 (87.27)	16 (100)	<0.01	0.01	0.03
In-hospital mortality within 90 days	4528 (35.70)	270 (31.51)	0.01	222 (30.37)	38 (34.55)	10 (62.50)	<0.01	0.80	0.03
**Solid Cancer with metastasis**									
	19,767	1013		840	156	17			
Admission to ICU	1727 (8.74)	218 (21.52)	<0.01	181 (21.55)	32 (20.51)	5 (29.41)	<0.01	<0.01	0.01
Severe complication during the inclusion stay	12,788 (64.69)	798 (78.78)	<0.01	671 (79.88)	113 (72.44)	14 (82.35)	<0.01	0.04	0.13
In-hospital mortality during the inclusion stay	7281 (36.83)	296 (29.22)	<0.01	236 (28.10)	55 (35.26)	5 (29.41)	<0.01	0.68	0.53
Severe complication within 90 days	14,015 (70.90)	851 (84.01)	<0.01	714 (85)	123 (78.85)	14 (82.35)	<0.01	0.03	0.43
In-hospital mortality within 90 days	9570 (48.41)	378 (37.31)	<0.01	305 (36.31)	67 (42.95)	6 (35.29)	<0.01	0.17	0.28
**Solid Cancer with localized tumor**									
	17,381	1390		1133	231	26			
Admission to ICU	2256 (12.98)	425 (30.58)	<0.01	349 (30.80)	66 (28.57)	10 (38.46)	<0.01	<0.01	<0.01
Severe complication during the inclusion stay	11,836 (68.10)	1088 (78.27)	<0.01	890 (78.55)	178 (77.06)	20 (76.92)	<0.01	<0.01	0.33
In-hospital mortality during the inclusion stay	4234 (24.36)	271 (19.5)	<0.01	221 (19.51)	43 (18.61)	7 (26.92)	<0.01	0.04	0.76
Severe complication within 90 days	12,833 (73.83)	1143 (82.23)	<0.01	930 (82.08)	190 (82.25)	23 (88.46)	<0.01	<0.01	0.09
In-hospital mortality within 90 days	5279 (30.37)	333 (23.96)	<0.01	274 (24.18)	51 (22.08)	8 (30.77)	<0.01	0.01	0.96

**Table 4:** Effect of obesity by type of cancer on the different outcomes, among COVID-19 cancer patients.

	In-Hospital Mortality during the Stay *	Severe Complications during the Stay *	Intensive Care Support during the Stay *	In-Hospital Mortality within 90 Days **	Severe Complications within 90 Days ***
	OR [95% CI]	OR [95% CI]	OR [95% CI]	HR [95% CI]	HR [95% CI]
All cancer	0.783 [0.719–0.852]	1.682 [1.531–1.847]	2.130 [1.952–2.323]	0.791 [0.741–0.844]	1.117 [1.094–1.139]
Hematological cancer	0.977 [0.831–1.148]	1.728 [1.424–2.096]	1.909 [1.631–2.233]	0.929 [0.820–1.053]	1.093 [1.053–1.134]
Solid Cancer with metastasis	0.733 [0.636–0.844]	1.791 [1.521–2.108]	2.225 [1.877–2.639]	0.762 [0.687–0.846]	1.153 [1.113–1.195]
Solid cancer with localized tumor	0.814 [0.705–0.939]	1.592 [1.378–1.840]	2.053 [1.791–2.354]	0.827 [0.738–0.925]	1.101 [1.067–1.137]

OR = Odds ratio; HR = Hazard ratio; CI = confidence interval * Logistic model adjusted on age class, sex, chemotherapy, dementia, heart failure, chronic respiratory disease, cirrhosis, diabetes, deficiency anemia and pulmonary bacterial infection; ** Cox model adjusted on age class, sex, dementia, heart failure, chronic respiratory disease, cirrhosis, diabetes, deficiency anemia and pulmonary bacterial infection; *** Fine & Gray model adjusted on age class, sex, dementia, heart failure, chronic respiratory disease, cirrhosis, diabetes, deficiency anemia and pulmonary bacterial infection.

**Table 5:** Effect of obesity according to its severity and by type of cancer on the different outcomes, among COVID-19 cancer patients.

	In-Hospital Mortality during the Stay *	Severe Complications during the Stay *	Intensive Care Support during the Stay *	In-Hospital Mortality within 90 Days **	Severe Complications within 90 Days ***
	OR [95% CI]	OR [95% CI]	OR [95% CI]	HR [95% CI]	HR [95% CI]
All cancer					
Standard obesity	0.750 [0.684–0.823]	1.714 [1.547–1.899]	2.136 [1.944–2.347]	0.772 [0.719–0.829]	1.118 [1.095–1.143]
Morbid obesity	0.914 [0.745–1.121]	1.433 [1.138–1.804]	2.063 [1.674–2.543]	0.860 [0.734–1.008]	1.091 [1.039–1.146]
Massive obesity	1.401 [0.806–2.434]	2.796 [1.320–5.922]	2.378 [1.353–4.179]	1.178 [0.789–1.759]	1.251 [1.113–1.406]
Hematological cancer					
Standard obesity	0.915 [0.768–1.090]	1.599 [1.302–1.963]	1.880 [1.587–2.226]	0.887 [0.774–1.016]	1.082 [1.040–1.127]
Morbid obesity	1.247 [0.819–1.899]	2.462 [1.406–4.312]	2.247 [1.505–3.353]	1.073 [0.778–1.480]	1.137 [1.040–1.243]
Massive obesity	3.094 [1.117–8.570]		1.197 [0.409–3.503]	2.202 [1.182–4.100]	1.303 [1.135–1.497]
Solid Cancer with metastasis					
Standard obesity	0.690 [0.590–0.807]	1.954 [1.632–2.341]	2.260 [1.880–2.717]	0.734 [0.654–0.824]	1.175 [1.132–1.221]
Morbid obesity	0.991 [0.709–1.385]	1.086 [0.735–1.607]	1.982 [1.301–3.019]	0.926 [0.727–1.179]	1.035 [0.941–1.139]
Massive obesity	0.808 [0.283–2.311]	2.482 [0.668–9.218]	2.742 [0.912–8.246]	0.757 [0.340–1.687]	1.162 [0.89–1.517]
Solid Cancer with localized tumor					
Standard obesity	0.793 [0.678–0.928]	1.617 [1.380–1.895]	2.067 [1.781–2.400]	0.821 [0.725–0.928]	1.096 [1.058–1.134]
Morbid obesity	0.863 [0.613–1.215]	1.446 [1.028–2.035]	1.881 [1.377–2.570]	0.820 [0.622–1.083]	1.112 [1.034–1.197]
Massive obesity	1.531 [0.618–3.794]	1.847 [0.690–4.948]	3.132 [1.366–7.181]	1.183 [0.591–2.370]	1.283 [1.065–1.546]

OR = Odds ratio; HR = Hazard ratio; CI = confidence interval; * Logistic model adjusted on age class, sex, chemotherapy, dementia, heart failure, chronic respiratory disease, cirrhosis, diabetes, deficiency anemia and pulmonary bacterial infection; ** Cox model adjusted on age class, sex, dementia, heart failure, chronic respiratory disease, cirrhosis, diabetes, deficiency anemia and pulmonary bacterial infection; *** Fine & Gray model adjusted on age class, sex, dementia, heart failure, chronic respiratory disease, cirrhosis, diabetes, deficiency anemia and pulmonary bacterial infection.

## Data Availability

The PMSI database was transmitted by the national agency for the management of hospitalization data. The use of these data by our department was approved by the National Committee for data protection. We are not allowed to transmit these data. PMSI data are available for researchers who meet the criteria for access to these French confidential data (this access is submitted to the approval of the National Committee for data protection) from the national agency for the management of hospitalization (ATIH—Agence technique de l’information sur l’hospitalisation, 117 boulevard Marius Vivier Merle, 69329 Lyon Cedex 03, France).

## Supplementary Materials

The following supporting information can be downloaded at: https://www.mdpi.com/article/10.3390/cancers14225660/s1, Table S1: ICD-10 codes used for identification of tumor subtypes; Table S2: ICD-10 codes used for obesity; Table S3: Admission to ICU, severe complication and in-hospital mortality of non COVID-19 cancer patients by type of cancer and obesity; Table S4: Effect of obesity by type of cancer on the different outcomes, among non COVID-19 cancer patients; Table S5: Effect of obesity according to its severity and by type of cancer on the different outcomes, among non COVID-19 cancer patients.
