# Supplementary material for: Effect of Obesity among Hospitalized Cancer Patients with or without COVID-19 on a National Level
Source: Cancers (Basel). 2022 Nov 17;14(22):5660. doi: 10.3390/cancers14225660 (PMC9688770; doi:10.3390/cancers14225660)
Supplement: Supplementary file 1 [file cancers-14-05660-s001.zip › cancers-1971252-supplementary.pdf]

Table S1. Characteristics of non-COVID-19 cancer patients by obesity.

	No obesity (1)	Obesity (2)	p-value (1 vs 2)	Standard obesity (3)	Morbid obesity (4)	Massive obesity (5)	p-value (1 vs 3)	p-value (1 vs 4)	p-value (1 vs 5)
**N**	892774	47035		39138	6915	982			
**Men, n(%)**	494171 (55.35)	23431 (49.82)	<0.01	20872 (53.33)	2345 (33.91)	214 (21.79)	<0.01	<0.01	<0.01
**Age, mean (std)**	68.74 (14.78)	66.95 (12.20)	<0.01	67.43 (12.14)	64.82 (12.27)	62.55 (11.69)	<0.01	<0.01	<0.01
**Age group (years)**			<0.01				<0.01	<0.01	<0.01
≤40	38123 (4.27)	1477 (3.14)		1122 (2.87)	308 (4.45)	47 (4.79)			
41-50	53745 (6.02)	3001 (6.38)		2343 (5.99)	555 (8.03)	103 (10.49)			
51-80	605181 (67.79)	36756 (78.15)		30470 (77.85)	5498 (79.51)	788 (80.24)			
81-90	161624 (18.10)	5294 (11.26)		4727 (12.08)	527 (7.62)	40 (4.07)			
>90	34101 (3.82)	507 (1.08)		476 (1.22)	27 (0.39)	4 (0.41)			
**Chemotherapy, n(%)**	260703 (29.20)	9254 (19.67)	<0.01	7739 (19.77)	1353 (19.57)	162 (16.50)	<0.01	<0.01	<0.01
**Comorbidities, n(%)**									
Hypertension	212207 (23.77)	23589 (50.15)	<0.01	19693 (50.32)	3426 (49.54)	470 (47.86)	<0.01	<0.01	<0.01
Dementia	18903 (2.12)	510 (1.08)	<0.01	439 (1.12)	61 (0.88)	10 (1.02)	<0.01	<0.01	0.02
HIV	2344 (0.26)	79 (0.17)	<0.01	69 (0.18)	9 (0.13)	1 (0.10)	<0.01	0.03	0.53
Heart failure	31778 (3.56)	2621 (5.57)	<0.01	2046 (5.23)	484 (7)	91 (9.27)	<0.01	<0.01	<0.01
Chronic respiratory disease	8124 (0.91)	1206 (2.56)	<0.01	798 (2.04)	326 (4.71)	82 (8.35)	<0.01	<0.01	<0.01
Chronic kidney disease	37915 (4.25)	3537 (7.52)	<0.01	2900 (7.41)	548 (7.92)	89 (9.06)	<0.01	<0.01	<0.01
Cirrhosis	17076 (1.91)	1651 (3.51)	<0.01	1354 (3.46)	260 (3.76)	37 (3.77)	<0.01	<0.01	<0.01
Diabetes	99028 (11.09)	14377 (30.57)	<0.01	11597 (29.63)	2424 (35.05)	356 (36.25)	<0.01	<0.01	<0.01
Peripheral vascular disease	26972 (3.02)	2156 (4.58)	<0.01	1935 (4.94)	202 (2.92)	19 (1.93)	<0.01	0.63	0.05
Dyslipidemia	43087 (4.83)	5852 (12.44)	<0.01	5070 (12.95)	696 (10.07)	86 (8.76)	<0.01	<0.01	<0.01
Deficiency Anemia	32308 (3.62)	1991 (4.23)	<0.01	1611 (4.12)	314 (4.54)	66 (6.72)	<0.01	<0.01	<0.01
COPD	39392 (4.41)	3791 (8.06)	<0.01	3123 (7.98)	583 (8.43)	85 (8.66)	<0.01	<0.01	<0.01
Pulmonary bacterial infection	12418 (1.39)	712 (1.51)	0.03	552 (1.41)	128 (1.85)	32 (3.26)	0.75	<0.01	<0.01
**Outcomes, n(%)**									
Admission to ICU	56365 (6.31)	4903 (10.42)	<0.01	3922 (10.02)	816 (11.80)	165 (16.80)	<0.01	<0.01	<0.01
Severe complication during the inclusion stay	283502 (31.76)	18412 (39.15)	<0.01	14932 (38.15)	2973 (42.99)	507 (51.63)	<0.01	<0.01	<0.01
In-hospital mortality during the inclusion stay	65751 (7.36)	2125 (4.52)	<0.01	1608 (4.11)	412 (5.96)	105 (10.69)	<0.01	<0.01	<0.01
Severe complication within 90 days	369908 (41.43)	21989 (46.75)	<0.01	17861 (45.64)	3543 (51.24)	585 (59.57)	<0.01	<0.01	<0.01
In-hospital mortality within 90 days	120698 (13.52)	3993 (8.49)	<0.01	3107 (7.94)	736 (10.64)	150 (15.27)	<0.01	<0.01	0.11

Table S2. Type of cancer by obesity among non-COVID-19 cancer patients.

	No obesity (1)	Obesity (2)	p-value (1 vs 2)	Standard obesity (3)	Morbid obesity (4)	Massive obesity (5)	p-value (1 vs 3)	p-value (1 vs 4)	p-value (1 vs 5)
**N**	892774	47035		39138	6915	982			
Hematological cancer, n(%)	93329 (10.45)	3994 (8.49)	<0.01	3235 (8.27)	651 (9.41)	108 (11)	<0.01	<0.01	0.58
Solid Cancer with metastasis, n(%)	265076 (29.69)	11579 (24.62)	<0.01	9603 (24.54)	1743 (25.21)	233 (23.73)	<0.01	<0.01	<0.01
Solid Cancer with localized tumor, n(%)	534369 (59.85)	31462 (66.89)	<0.01	26300 (67.20)	4521 (65.38)	641 (65.27)	<0.01	<0.01	<0.01

Table S3. Admission to ICU, severe complication and in-hospital mortality of non COVID-19 cancer patients by type of cancer and obesity.

**Hematological cancer**	**No obesity (1)**	**Obesity (2)**	**p-value (1 vs 2)**	**Standard obesity (3)**	**Morbid obesity (4)**	**Massive obesity (5)**	**p-value (1 vs 3)**	**p-value (1 vs 4)**	**p-value (1 vs 5)**
	93329	3994		3235	651	108			
Admission to ICU	15023 (16.10)	888 (22.23)	<0.01	713 (22.04)	149 (22.89)	26 (24.07)	<0.01	<0.01	0.02
Severe complication during the inclusion stay	39511 (42.34)	2216 (55.48)	<0.01	1758 (54.34)	393 (60.37)	65 (60.19)	<0.01	<0.01	<0.01
In-hospital mortality during the inclusion stay	7614 (8.16)	293 (7.34)	0.06	221 (6.83)	61 (9.37)	11 (10.19)	<0.01	0.26	0.44
Severe complication within 90 days	49419 (52.95)	2544 (63.70)	<0.01	2022 (62.50)	448 (68.82)	74 (68.52)	<0.01	<0.01	<0.01
In-hospital mortality within 90 days	13126 (14.06)	468 (11.72)	<0.01	360 (11.13)	92 (14.13)	16 (14.81)	<0.01	0.96	0.82
**Solid Cancer with metastasis**	**No obesity (1)**	**Obesity (2)**	**p-value (1 vs 2)**	**Standard obesity (3)**	**Morbid obesity (4)**	**Massive obesity (5)**	**p-value (1 vs 3)**	**p-value (1 vs 4)**	**p-value (1 vs 5)**
	265076	11579		9603	1743	233			
Admission to ICU	14131 (5.33)	1187 (10.25)	<0.01	970 (10.10)	180 (10.33)	37 (15.88)	<0.01	<.001	<0.01
Severe complication during the inclusion stay	94587 (35.68)	4955 (42.79)	<0.01	3998 (41.63)	816 (46.82)	141 (60.52)	<0.01	<0.01	<0.01
In-hospital mortality during the inclusion stay	37211 (14.04)	1004 (8.67)	<0.01	765 (7.97)	193 (11.07)	46 (19.74)	<0.01	<0.01	0.01
Severe complication within 90 days	123224 (46.49)	6018 (51.97)	<0.01	4863 (50.64)	992 (56.91)	163 (69.96)	<0.01	<0.01	<0.01
In-hospital mortality within 90 days	67324 (25.40)	1905 (16.45)	<0.01	1491 (15.53)	349 (20.02)	65 (27.90)	<0.01	<0.01	0.38
**Solid Cancer with localized tumor**	**No obesity (1)**	**Obesity (2)**	**p-value (1 vs 2)**	**Standard obesity (3)**	**Morbid obesity (4)**	**Massive obesity (5)**	**p-value (1 vs 3)**	**p-value (1 vs 4)**	**p-value (1 vs 5)**
	534369	31462		26300	4521	641			
Admission to ICU	27211 (5.09)	2828 (8.99)	<0.01	2239 (8.51)	487 (10.77)	102 (15.91)	<0.01	<0.01	<0.01
Severe complication during the inclusion stay	149404 (27.96)	11241 (35.76)	<0.01	9176 (34.89)	1764 (39.02)	301 (46.96)	<0.01	<0.01	<0.01
In-hospital mortality during the inclusion stay	20926 (3.92)	828 (2.63)	<0.01	622 (2.37)	158 (3.49)	48 (7.49)	<0.01	0.15	<0.01
Severe complication within 90 days	197265 (36.92)	13427 (42.68)	<0.01	10976 (41.73)	2103 (46.52)	348 (54.29)	<0.01	<0.01	<0.01
In-hospital mortality within 90 days	40248 (7.53)	1620 (5.15)	<0.01	1256 (4.78)	295 (6.53)	69 (10.76)	<0.01	0.01	<0.01

Table S4. Effect of obesity by type of cancer on the different outcomes, among non COVID-19 cancer patients.

	**In-hospital mortality during the stay ***	**Severe complications during the stay ***	**Intensive care support during the stay ***	**In-hospital mortality within 90 days ****	**Severe complications within 90 days *****
	**OR [95% CI]**	**OR [95% CI]**	**OR [95% CI]**	**HR [95% CI]**	**HR [95% CI]**
**All cancer**	0.620 [0.592-0.649]	1.340 [1.311-1.370]	1.530 [1.481-1.580]	0.648 [0.628-0.669]	1.116 [1.103-1.128]
**Hematological cancer**	0.877 [0.773-0.994]	1.491 [1.385-1.604]	1.396 [1.288-1.514]	0.830 [0.756-0.912]	1.148 [1.115-1.183]
**Solid Cancer with metastasis**	0.588 [0.550-0.629]	1.261 [1.208-1.316]	1.693 [1.586-1.807]	0.638 [0.610-0.668]	1.093 [1.070-1.117]
**Solid Cancer with localized tumor**	0.676 [0.629-0.728]	1.695 [1.357-1.434]	1.515 [1.452-1.581]	0.692 [0.658-0.728]	1.137 [1.120-1.155]

* Logistic model adjusted on age class, sex, chemotherapy, dementia, heart failure, chronic respiratory disease, cirrhosis, diabetes, deficiency anemia and pulmonary bacterial infection; ** Cox model adjusted on age class, sex, dementia, heart failure, chronic respiratory disease, cirrhosis, diabetes, deficiency anemia and pulmonary bacterial infection; *** Fine & Gray model adjusted on age class, sex, dementia, heart failure, chronic respiratory disease, cirrhosis, diabetes, deficiency anemia and pulmonary bacterial infection.

Table S5. Effect of obesity according to its severity and by type of cancer on the different outcomes, among non COVID-19 cancer patients.

	**In-hospital mortality during the stay ***	**Severe complications during the stay ***	**Intensive care support during the stay ***	**In-hospital mortality within 90 days ****	**Severe complications within 90 days *****
	**OR [95% CI]**	**OR [95% CI]**	**OR [95% CI]**	**HR [95% CI]**	**HR [95% CI]**
**All cancer**					
Standard obesity	0.559 [0.531-0.589]	1.289 [1.258-1.320]	1.488 [1.436-1.541]	0.603 [0.581-0.625]	1.090 [1.077-1.104]
Morbid obesity	0.852 [0.769-0.944]	1.581 [1.495-1.671]	1.655 [1.532-1.787]	0.839 [0.780-0.902]	1.229 [1.195-1.264]
Massive obesity	1.649 [1.336-2.035]	2.190 [1.890-2.538]	2.334 [1.954-2.788]	1.247 [1.062-1.464]	1.348 [1.246-1.459]
**Hematological cancer**					
Standard obesity	0.798 [0.691-0.920]	1.415 [1.305-1.533]	1.417 [1.296-1.550]	0.775 [0.697-0.861]	1.129 [1.093-1.167]
Morbid obesity	1.236 [0.940-1.626]	1.892 [1.584-2.261]	1.283 [1.058-1.556]	1.080 [0.879-1.328]	1.235 [1.154-1.321]
Massive obesity	1.457 [0.769-2.762]	1.810 [1.162-2.818]	1.507 [0.952-2.386]	1.195 [0.731-1.953]	1.202 [1.013-1.427]
**Solid Cancer with metastasis**					
Standard obesity	0.531 [0.492-0.572]	1.203 [1.148-1.260]	1.696 [1.579-1.821]	0.594 [0.565-0.626]	1.067 [1.042-1.092]
Morbid obesity	0.816 [0.700-0.950]	1.513 [1.359-1.685]	1.602 [1.363-1.884]	0.831 [0.748-0.924]	1.207 [1.144-1.273]
Massive obesity	1.614 [1.157-2.253]	2.532 [1.880-3.411]	2.231 [1.537-3.239]	1.193 [0.935-1.522]	1.353 [1.125-1.626]
**Solid Cancer with localized tumor**					
Standard obesity	0.603 [0.555-0.656]	1.350 [1.310-1.391]	1.452 [1.385-1.522]	0.638 [0.603-0.675]	1.111 [1.093-1.130]
Morbid obesity	0.937 [0.793-1.106]	1.608 [1.498-1.726]	1.749 [1.583-1.933]	0.906 [0.808-1.017]	1.257 [1.212-1.304]
Massive obesity	2.207 [1.616-3.013]	2.190 [1.817-2.639]	2.462 [1.958-3.096]	1.602 [1.264-2.030]	1.400 [1.276-1.537]

* Logistic model adjusted on age class, sex, chemotherapy, dementia, heart failure, chronic respiratory disease, cirrhosis, diabetes, deficiency anemia and pulmonary bacterial infection; ** Cox model adjusted on age class, sex, dementia, heart failure, chronic respiratory disease, cirrhosis, diabetes, deficiency anemia and pulmonary bacterial infection; *** Fine & Gray model adjusted on age class, sex, dementia, heart failure, chronic respiratory disease, cirrhosis, diabetes, deficiency anemia and pulmonary bacterial infection.
